# Patient-Derived Stem Cell Models in *SPAST* HSP: Disease Modelling and Drug Discovery

**DOI:** 10.3390/brainsci8080142

**Published:** 2018-07-31

**Authors:** Gautam Wali, Carolyn M. Sue, Alan Mackay-Sim

**Affiliations:** 1 Department of Neurogenetics, Kolling Institute of Medical Research, The University of Sydney, Sydney, NSW 2065, Australia; gautam.wali@sydney.edu.au (G.W.); carolyn.sue@sydney.edu.au (C.M.S.); 2 Griffith Institute for Drug Discovery, Griffith University, Brisbane, QLD 4111, Australia

**Keywords:** neurodegeneration, microtubule, organelle transport, peroxisome, spastic paraplegia, SPG4, *SPAST*, spastin

## Abstract

Hereditary spastic paraplegia is an inherited, progressive paralysis of the lower limbs first described by Adolph Strümpell in 1883 with a further detailed description of the disease by Maurice Lorrain in 1888. Today, more than 100 years after the first case of HSP was described, we still do not know how mutations in HSP genes lead to degeneration of the corticospinal motor neurons. This review describes how patient-derived stem cells contribute to understanding the disease mechanism at the cellular level and use this for discovery of potential new therapeutics, focusing on *SPAST* mutations, the most common cause of HSP.

## 1. Genetics of Hereditary Spastic Paraplegia (HSP)

Hereditary spastic paraplegia (HSP) is a neurological disorder in which the corticospinal motor neurons degenerate [1,2]. It is one of the most genetically heterogeneous disorders. To date, more than 84 HSP loci have been mapped and 67 HSP genes identified [3]. HSP involves all modes of inheritance including autosomal dominant, autosomal recessive, and X-linked recessive patterns [4,5], however, inheritance is most commonly autosomal dominant [4,5,6]. *SPAST* gene mutation accounts for up to 40% of autosomal-dominant mutations, making it the most commonly mutated gene in this family of disease [6,7].

Spastin, encoded by *SPAST*, is a protein of the AAA (ATPase associated with diverse cellular activities) protein family [8,9]. Spastin regulates multiple cellular functions, including microtubule dynamics [10], shaping endoplasmic reticulum [11], and regulating lipid droplet metabolism [12]. Our review focuses on the microtubule dynamics regulation function of spastin. 

## 2. Radiology of HSP

In recent studies, cerebral white matter alterations in HSP patients were quantified using diffusion tensor imaging (DTI), a widely used magnetic resonance imaging technique. DTI measures the anisotropic flow of water in white matter tracts [13]. White matter diffusion patterns can be influenced by factors such as orientation of the axons in the tract, axon damage, or by the degree of myelination. The common DTI indices are: fractional anisotropy, a measure of sensitivity to changes in orientation of axons along the tracts; mean diffusivity, a measure of magnitude of water diffusion and the presence of obstacles to diffusion [14,15], and radial diffusion, used to differentiate axonal injury from demyelination [13]. The most common radiological changes in a variety of HSP gene mutations are alterations in the corticospinal tract (70% of all studies, 71% of *SPAST* studies) and corpus callosum (80% of all studies, 86% of *SPAST* studies) [16,17,18,19,20,21,22,23,24,25]. Loss or damage to axons in the corticospinal tract are consistent with the motor symptoms of the disease, although white matter disturbances are not confined to the corticospinal tract and corpus callosum with involvement at the whole brain level, frontal and temporal lobes, cerebellum, and other regions. These observations were made with a variety of DTI techniques, fractional anisotropy being the commonly quantified measure. It is worth noting that the extent of affected regions may be underestimated due to the limits of DTI sensitivity [26]. These changes are consistent with widely distributed axonal damage in the white matter of the brain, including the corticospinal tract, which contains the axons of the motor neurons projecting to the lower motor neurons in the distal spinal cord, whose degeneration is responsible for the clinical manifestations of HSP. There is evidence for correlation between radiological findings and disease severity and duration [16,17,19,24]. Future clinical investigations could consider examining late-stage *SPAST* HSP patients for evidence of non-motor manifestations that are seen in patients with other HSP mutations [27]. The consistency of the MRI findings suggests that low fractional anisotropy in corticospinal tract and corpus callosum may be useful clinical markers and could be potential biomarkers for future clinical trials. A major aim for researchers is to understand the cellular mechanisms leading to degeneration of the corticospinal tract.

## 3. Histological Findings in HSP Patients and Animal Models

Post-mortem studies of six HSP patients showed the loss of small and large fibres of the corticospinal tract leading to significant reductions in axon numbers and tract volumes throughout the spinal cord and into the medulla [28]. These cases also showed significant losses in axon numbers and volumes in the ascending sensory tracts. At the cellular level, defects in HSP patient cells have been observed mainly in neurons [29,30,31,32]. Axonal swellings and abnormal organelle distribution have been the hallmarks of axonal defects in HSP patients and in mouse models.

### 3.1. HSP Patients

Post-mortem analysis of spinal cord sections at the cervical and lumbar level of two *SPAST* HSP patients carrying missense and frameshift mutations had axonal swellings in the descending axons of the corticospinal tract and dorsal column of the spinal cord [32]. The axonal swellings were filled with neurofilaments and mitochondria. Immunohistochemistry study of three *SPAST* HSP cases showed altered mitochondrial distribution in the cell body of spinal cord lower and upper motor neurons, with mitochondria being restricted to the periphery of the neuronal soma, in contrast to uniformly distributed mitochondria in control cells [31].

### 3.2. Mouse Models

*SPAST* mouse models do not exhibit the characteristic corticospinal degeneration, but retain the neuronal defects of axonal swellings and altered organelle distribution. In a mouse model with exon 5–7 deletion in *SPAST* (leading to the lack of the AAA domain in spastin), spinal cord sections of 12 and 24 months homozygous mouse at the cervical and lumbar level did not show any obvious degeneration of the corticospinal or dorsal columns [33]. Spinal cord sections of the same homozygous mouse model at the cervical and lumbar levels of four months, 12 months, and 24 months old mouse showed axonal swellings. In contrast, heterozygous mouse had few axonal swellings comparable to control mouse. Comparison of axonal swellings between heterozygous and homozygous 12 months mutant mouse showed 11-fold fewer axonal swellings in heterozygous mouse. The axonal swelling defect in the homozygous model increased linearly with the mouse age, consistent with progressive HSP seen clinically although human patients have heterogeneous *SPAST* mutations [33].

Homozygous mouse spinal cord sections also showed abnormal accumulation of neurofilaments and cellular organelles in the axonal swellings, including mitochondria and peroxisomes [33], as observed in HSP patient spinal cord sections [32]. In another mouse model, with splice donor site mutation in Exon 7 of *SPAST*, spinal cord sections of three month old mice with heterozygous and homozygous mutations mouse there were axonal swellings in the white matter [32]. The study did not present their data for wild-type/control mouse, making it unclear whether the axonal phenotype was specific to *SPAST* mutant mouse or was present, regardless. Although the two mouse studies agree on the axonal defect of axonal swellings in HSP, there were inconsistencies. In the former model, the swellings were specific to distal regions of the axons [33], whereas in the latter model, the swellings occurred randomly along the length of the axonal compartment [32].

In summary, although the mouse models show swelling defects in corticospinal axons that are similar to corticospinal tract axons in *SPAST* HSP patients [32], there is no evidence for the degeneration of the corticospinal tract [28]. This suggests that axonal defects are sufficient for the motor behaviour deficits in the mouse [33] without requiring significant die-back of corticospinal axons. This supports the MRI evidence indicating that the loss of corticospinal axons is a later stage phenomenon. This has implications for the success of future therapeutics, which may be most effective when given early in the disease progression.

### 3.3. Other Animal Models

Functional studies in Drosophila and zebrafish have shown that spastin function is essential for motor neurons [8,34,35,36]. Knockdown of spastin in zebrafish model, severely impaired axon outgrowth of spinal motor neuron axons with defects in neuronal connectivity, and a disrupted network of stabilised microtubules [35]. In Drosophila, the loss of spastin caused loss of synaptic area at the neuromuscular junction. At the same synapse, loss of spastin increased the level of acetylated α-tubulin (a marker of stabilised microtubules), whereas increased spastin reduced the level of acetylated α-tubulin [36]. In another study of spastin null mutant Drosophila, there was reduced expression of futsch (another marker of stabilised microtubules) in the synaptic boutons at the neuromuscular junction [8]. Thus, both studies agree that a loss of spastin in neuromuscular synaptic boutons leads to changes in stabilised microtubules, the direction of the changes contradict: increased [36] vs. decreased [8]. These Drosophila models do not show any axonal degeneration, axonal swellings, or organelle distribution defects that are seen in HSP patients.

Genetic manipulation in animal models can help in our understanding of the molecular consequences of spastin mutations, but it is still a challenge to understand the disease mechanism in humans. Perhaps this is because animal models, unlike human patients, are genetically homogeneous and there are major species-specific differences at all levels (anatomy, physiology, genetics, behaviour, cell biology). Genetically modified animal models invariably fail to fully replicate human disease deficits and when used for drug development produce, therapeutics that work well in these animal models, work poorly in humans, which is a common problem in neuroscience therapeutic development [37,38,39]. An alternative to animal models in HSP is to use patient-derived brain tissue or cell models that represent the genetic and phenotypic diversity of the patient population. Brain tissue obtained post mortem poses challenges because of the difficulty in obtaining fresh brain tissue at death, the absence of ability to study progression of the disease when the samples are confined to the end stage, and the lack of ability to investigate genetic and molecular pathologies in living cells. Cells from HSP patients are potential disease models that additionally provide early toxicity testing during the drug discovery process. The challenge is to find appropriate, easily accessible cells. Accessible non-neuronal cells like fibroblasts may not show strong patient-control differences for understanding the cellular basis of neurological diseases [40]. Patient-derived stem cells are filing this void.

## 4. Patient-Derived Stem Cell Models in HSP

### 4.1. Induced Pluripotent Stem Cells

Induced pluripotent stem (iPS) cells [41] are generated by genetically reprogramming accessible somatic cells, most often skin fibroblast cells. iPS cells can propagate indefinitely in vitro and can give rise to any cell type of the body, including neurons. iPS cells resemble human embryonic stem cells in aspects of proliferation, morphology, gene expression profiles, pluripotency genes, and differentiation ability. Patient-derived iPS cells have been used for disease modelling of numerous neurological disorders, including Parkinson’s disease [42], Amyotrophic lateral sclerosis [43], Spinal muscular atrophy [44], and Alzheimer’s disease [45], and they have been used to screen for drugs [46]. To study the underlying disease mechanism of the degenerating corticospinal tract in HSP, it would be ideal to generate neurons that make up the corticospinal tract i.e., the cortical motor pyramidal neurons. Relevant published protocols are available for this [47]. But, as axonal loss in HSP is not confined to the corticospinal tract upon which diagnosis is dependent, but is more widespread. This includes regions, such as the corpus callosum, internal capsule, external capsule, and others (refer “Pathology of HSP” section), for which specific differentiation protocols are not yet available.

Two studies of neurons generated from *SPAST* HSP patient-derived iPS cells have reported axonal swellings and axonal transport deficits. Glutamatergic neurons from one iPS cell line carrying the heterozygous *SPAST* c.683-iG > T mutation showed increased axonal swellings and altered mitochondria transport with reductions in the percentage of microtubule-dependent mitochondria, reduced retrograde, and unchanged anterograde transport [29]. Another study of glutamatergic neurons that were generated from two iPS cell lines carrying the heterozygous *SPAST* mutation c.1684C > T showed alterations in neuron morphology with a reduced number and length of neurites; increased axonal swellings and altered mitochondria transport with reduced retrograde and increased anterograde transport [29,30]. Although both of these studies showed that *SPAST* iPS-derived neurons can identify HSP disease-associated neuronal deficits, they do not suggest the mechanism of how the observed axonal defects could lead to axonal degeneration in HSP patients. 

iPS cells hold great potential use in disease modelling and drug screening of human diseases because they provide an unlimited source of tissue-specific cells, but there are still technical challenges for their widespread use as disease models [48]. Development of non-integrating vectors for reprogramming have obviated some of the off-target effects of early methods of iPS genetic reprogramming, but reprogramming methods are still inefficient, laborious, expensive, and time-consuming posing real challenges to application in multiple labs and in multiple patients. As a consequence of the reprogramming process and prolonged cell culture, iPS cells can develop a wide range of variations, including aneuploidy and chromosomal aberrations, single nucleotide variations, and sub-chromosomal copy number variations [49,50,51]. Differentiation of iPS cells into neurons can be problematic: efficiency was low and variable between iPS cell lines that were generated by the same reprogramming method, different reprogramming methods, and between iPS cell lines that are generated from the same parental fibroblast cell line [52]. With time and cost limiting the numbers of disease-specific iPS cell studies, such technical issues in reprogramming and neuronal differentiation are potential confounds that need to be resolved when trying to identify subtle disease-specific defects in patient iPS-based cell models. 

### 4.2. Adult Olfactory Stem Cells

Patient-derived olfactory cells and tissues are increasingly used for investigating neurodegenerative and neuropsychiatric diseases for which they have certain advantages over iPS cell models [53]. The olfactory sensory neurons are continually regenerated throughout life from stem cells in the olfactory mucosa, which is the organ of smell in the nose, which is accessible by simple biopsy in most people under local anaesthetic [54]. Olfactory neurosphere-derived cells (ONS cells), are adult stem cells that are derived from the olfactory mucosa. These are a multipotent stem cell, able to make neurons and glia as well as many other cell types of the body (heart, liver, kidney, blood: [55]). ONS cells can be generated quickly from patient biopsies using standardised protocols [56]. They do not proliferate indefinitely but frozen stocks can be created and used for many years as a novel way of investigating brain diseases and for drug discovery using high throughput biology and automated screening to generate and test hypothesis [38,39]. ONS cells have revealed significant disease-specific phenotypes in schizophrenia, Parkinson’s disease, ataxia telangiectasia, epilepsy, autism, familial dysautonomia, and HSP [57,58,59,60,61,62,63,64]. Unlike iPS cells, ONS cells are not genetically reprogrammed and they are relatively easy to generate in vitro and cost-effective to generate and maintain [38]. Like iPS cells, neuronal differentiation of ONS cells is variable and not efficient [53,55], and there are no protocols for differentiating them into specific neuronal populations as there are for iPS cells, thus limiting the ability to use ONS-derived cells to investigate defined neurons, such as cortical neurons.

As cell models of HSP, we generated ONS cell lines from nine patients with heterozygous *SPAST* mutations and ten healthy controls. By comparing the differences between patient- and control-derived cells, we identified altered cell functions in patient cells and identified drugs that restored their function, thus making them potential new therapeutics for HSP. In discovery phase, an initial unbiased transcriptomics analysis showed that 57% of all mRNA transcripts were affected in patient cells suggesting that *SPAST* mutations cause major changes in cellular homeostasis [61]. Consistent with this hypothesis, gene ontology analysis of the dysregulated transcripts pointed to widespread alterations in the expression of multiple genes that are involved in microtubule formation and dynamics. Proteomic analysis revealed a 50% reduction in spastin in patient cells when compared to controls, indicating the link between gene haploinsufficiency and protein expression in *SPAST* patient cells. Further analysis of microtubule-associated proteins showed a 50% reduction in acetylated α-tubulin, a marker of stable microtubules and substrate of spastin, and a 150% increase in stathmin, a microtubule destabilising enzyme. This reduction in stabilised microtubules when spastin is reduced may be explained by increased expression of stathmin, as a consequence of homeostatic feedback regulation of microtubule dynamics.

Quantitative automated microscopic analysis confirmed the loss of stable microtubules throughout the patient cells and altered the distributions of peroxisomes (more numerous at the periphery of patient cells compared to control cells) and mitochondria (less numerous at the periphery), suggesting that organelle transport was impaired in patient cells [61]. Displacement of mitochondria was also seen in neuronal cell bodies in the HSP spinal cord [31]. Peroxisome transport was analysed by live-cell imaging of patient and control ONS cells: the average speeds of peroxisome transport was significantly reduced in patient cells [61].

Intracellular microtubule-dependent organelle transport is essential in neuronal axons that depend solely on microtubules for intracellular transport to and from their distally located synapses (up to 1 m in cortical neurons) [65]. We tested microtubule-dependency of peroxisome transport by differentiating ONS cells into immature neurons with long neurites that mimicked axons, comprising axial microtubules without the background of actin cytoskeleton that is found in cell bodies [62]. With this cytoarchitecture, the patient-control differences were amplified in the longer neuron-like cells when compared to smaller ONS cells (~50% reduction in mean speed compared to ~10% in ONS cells) [61,62]. Additionally, there was a 25% reduction in the numbers of peroxisomes in patient neurites as compared to control neurites and a relative reduction in the number of peroxisomes transported retrogradely [62]. For further evidence of microtubule-dependence, we separately analysed the transport speeds of the fast peroxisomes as they are known to be dependent on microtubules for their transport [66]. The speeds of the fast peroxisomes in patient-derived neurites were significantly less than in controls [62]. Peroxisome movement is saltatory, with short bursts of movement followed by rest periods. Peroxisome speeds are governed in part by the number and duration of these saltatory movements [66]. This was investigated by quantifying time-dependent movements of the fast peroxisomes. As there was no patient-control difference in number and duration of salutatory events in the microtubule-dependent fast peroxisomes, the speed reduction of the peroxisome population in patient cells is likely due to the reduced availability of stabilised microtubules in patient cells, rather than reduced ability of peroxisomes to attach and move along stabilised microtubules as a consequence of mutated spastin in patient cells [62]. This hypothesis was confirmed when stabilised microtubule content was raised pharmacologically to control levels, which restored peroxisome transport speeds to control levels [58].

Peroxisomes are important regulators of oxidative state in cells, especially the metabolism of hydrogen peroxide [67]. So, deficits in peroxisome transport could reduce cell viability due to oxidative stress. This was confirmed in *SPAST* patient ONS cells that are under oxidative stress when compared to control cells, and more sensitive to hydrogen peroxide [62]. In patient cells, there was a significant increase in the expression of 4HNE, a lipid peroxidation product, under baseline culture conditions and after exposure to hydrogen peroxide. Additionally, exposure to hydrogen peroxide was more toxic to patient cells when compared to control cells, as assessed by ATP production and cell viability assays. This is the first evidence of oxidative stress in *SPAST* HSP patient-derived cells and it links *SPAST* mutations with the induction of cell death via a reduction in stabilised microtubules. This mechanism was confirmed in patient cells when acetylated α-tubulin levels were restored pharmacologically to control levels and their oxidative response to hydrogen peroxide was also restored [62].

Peroxisomes and mitochondria interact to regulate responses to oxidative stress and altered biogenesis, dynamics, or metabolism in one organelle can affect the other [68]. Mitochondria contribute fundamentally to cellular homeostasis, including ATP production and dynamics, metabolism of reactive oxygen species, and calcium and lipid homeostasis. The major functions of peroxisomes include fatty acid α- and β-oxidation, ether lipid (esp. plasmalogen) biosynthesis, and reactive oxygen species homeostasis [67]. Therefore, in *SPAST* HSP, dysfunctions in microtubule-dependent transport of peroxisomes and mitochondria could have profound consequences for many cell functions, requiring dynamic homeostatic feedback. This may help to explain the need for the genome-scale changes that were observed in the transcriptome of *SPAST* patient cells [61]. Presumably, *SPAST* mutations would affect organelle transport in all cells of the body, but with more profound effects in long axons that depend on microtubules alone for organelle transport. In agreement with this, is evidence that *SPAST* patient muscle is morphologically normal, despite similarly large magnitude changes in the transcriptome and disruption of microtubule pathways [69].

Peroxisome and mitochondria gene mutations are associated with numerous developmental and other deficits in several neurological diseases [70,71,72,73], including HSP [74], and impaired mitochondrial and peroxisomal regulation of oxidative stress have been implicated in the aetiology of chronic conditions, such as inflammation, type 2 diabetes, neurodegeneration, and aging [75]. *SPG7* HSP patient-derived fibroblasts are more sensitive to hydrogen-peroxide with lower ATP production and viability when compared to control fibroblasts [76].

Neurons are particularly vulnerable to oxidative stress, related to the unique functional and structural organisation of neurons. Neurons have a very high requirement for oxygen for intracellular ion homoeostasis, which is essential for propagation of action potentials [77,78]. Neurons are enriched in polyunsaturated fatty acids that are primary targets of reactive oxygen species-induced oxidative damage [78]. Oxidative stress leading to an increase in 4HNE can also damage the blood brain barrier by increasing its permeability [79]. These features may explain why the long corticospinal neurons are more susceptible to compromised organelle transport in HSP, with injury starting distally in these long axons with progressive axon die-back along the corticospinal tract. This hypothesis fits well with the progressive clinical presentation of *SPAST* HSP patients whose initially moderate symptoms increase in severity over time as initial axon impairment leads increasingly to axon death and die-back.

### 4.3. Two Stem Cell Models Combined

Patient and control-derived ONS cells are relatively easy to generate and freeze/thaw in vitro, making them suitable for multiple high-through put assays for hypothesis generation and the discovery of disease-associated cell dysfunctions. ONS cells are neural progenitors but they are not neurons and so may not reveal neuron-specific dysfunctions resulting from disease-associated mutations. Our disease-modelling approach is to use patient- and control-derived ONS cells as discovery tools to generate hypothesis and to understand the domain of deficits that are associated with HSP mutations and to use this understanding to screen for possible therapeutic drug candidates. Specific hypotheses are then validated in neurons differentiated from patient-derived iPS cells, keeping those experiments to a minimum because they are more time-consuming to generate and expensive to maintain than ONS cells. We see this two-stage approach as the fastest and most inexpensive way to use patient-derived stem cells for drug discovery and clinical outcomes. We generated 11 iPS cell lines from three *SPAST* patients and three healthy controls and differentiated them into cortical neurons (unpublished data) [80]. Like ONS cells, *SPAST* patient-derived neurons had reduced spastin, reduced acetylated α-tubulin, reduced transport of peroxisomes, and were more vulnerable to hydrogen peroxide-induced oxidative stress demonstrated by increased axonal degeneration (unpublished data) [80].

### 4.4. Tubulin-Binding Drugs as Therapeutic Candidates

**Figure 1:**
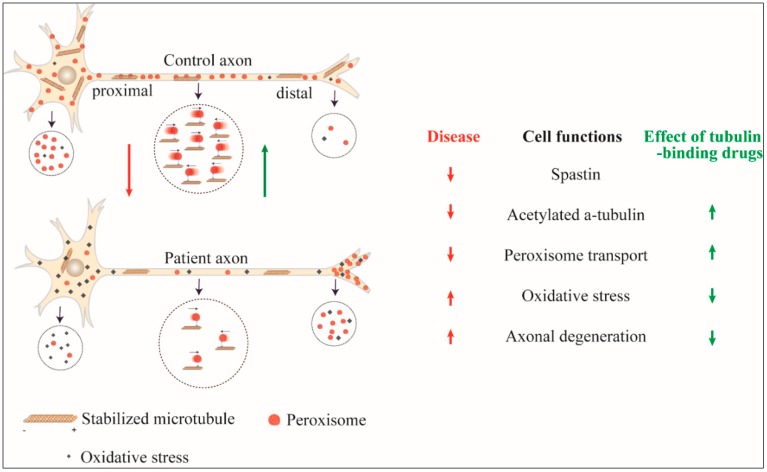
Effect of *SPAST* mutation and its reversal by tubulin-binding drugs. Axonal transport of peroxisomes is reduced in *SPAST* hereditary spastic paraplegia (HSP) cells with reduced retrograde transport leading to accumulation at the periphery, distal oxidative stress and axon degeneration. By increasing acetylated α-tubulin, tubulin-binding drugs restore peroxisome transport and the adverse consequences of the *SPAST* mutation.

*SPAST* patient-derived ONS cells and patient-derived neurons demonstrate reduced levels of stabilised microtubules, impaired peroxisome transport, increased oxidative stress, and sensitivity to hydrogen peroxide, leading to reduced cell viability and increased axon degeneration (Figure 1). To correct these deficits, we reasoned that it may not be necessary to correct the spastin insufficiency directly but it may only be required to correct the downstream homeostatic change in acetylated α-tubulin. Microtubule-dependent organelles are transported along microtubules of polymerised tubulin [81]. Tubulin normally undergoes various post-translational modifications [82], including acetylation of α-tubulin, which is considered to be a signal for the motor proteins to tether for organelle transport [81,82,83]. When considering this, restoring levels of acetylated α-tubulin in patient-derived cells would increase the tethering of peroxisomes and increase the numbers of microtubule transported peroxisomes and increase the average transport speeds of the peroxisome population. With this logic, we investigated tubulin-binding drugs for their effects on peroxisome transport in patient-derived ONS cells and neurons given their ability to disrupt microtubule dynamics in other cells: taxol and epothilones promote the assembly of microtubules [84,85], vinblastine inhibits assembly of microtubules [86] and noscapine alters the steady state dynamics of microtubule assembly [87]. Vinblastine reduced the number of axonal swellings in *SPAST* patient iPS cell-derived neurons [29]. Taxol and vinblastine also reduced axonal swellings in a *SPAST* mouse model [88]. 

Using automated high content imaging, we first identified low concentrations of four tubulin-binding drugs (taxol, vinblastine, epothilone D, and noscapine) that increased the levels of acetylated α-tubulin in patient ONS cells to levels in control ONS cells [58]. We then quantified the effects of these low doses of tubulin-binding drugs on peroxisome transport, demonstrating that they restored peroxisome transport in patient cells to control cell speeds by increasing the number of fast microtubule-dependent peroxisomes [58,62]. Epothilone D also reduced the sensitivity to hydrogen peroxide-induced oxidative stress [62]. Similar results were observed in neurons that were generated from patient-derived iPS cells: epothilone D and noscapine also rescued peroxisome axon transport deficits and ameliorated axonal degeneration induced by hydrogen peroxide (unpublished) [80]. These results confirm that impaired peroxisome transport in patient cells is a consequence of reduced levels of acetylated α-tubulin in patient cells, and this was evident in patients with a variety of different mutations in *SPAST*. They also identify tubulin-binding drugs as potential therapies for HSP (Table 1). Epothilone D and noscapine are permeant to the blood brain barrier, and are hence potentially able to reach the cortical motor neurons and their axons in patients.

Deficits in microtubule-associated proteins and impaired organelle transport are emerging as common mechanisms in several motor neuron diseases and neurodegenerative disorders [89,90,91], while chronic oxidative stress is seen as another mechanism for neuronal loss in neurodegenerative diseases [92,93]. Our evidence in patient-derived cells demonstrates a direct link between these mechanisms for peroxisome transport in HSP patients with a variety of *SPAST* mutations. This mechanism may also apply to mitochondria whose transport is impaired in other *SPAST* patient-derived cells [29,30].

The tubulin-binding drugs that we used in our studies have been “re-purposed” from cancer therapies and large doses have passed phase I clinical trials, demonstrating their safety. The drug doses in this study are much lower than used clinically [94] and did not show any cytotoxicity to patient-derived cells [58]. In progressing to clinical trial, epothilone D and noscapine are attractive candidates as they can readily cross the blood brain barrier and [95,96] in contrast to the taxol and vinblastine [97]. This “repurposing” of approved drugs can be important for rare neurological disorders, like HSP, as it reduces the time and costs involved in developing a new drug, making it unprofitable for pharmaceutical companies. 

## 5. Conclusions

With the many genes that can cause HSP, there is a real challenge in understanding the genetic and cellular mechanisms of disease and in devising individual therapies. It is not obvious from the causative mutated genes if there are any common cellular mechanisms that can be targeted for therapy. These might be revealed by the use of patient-derived stem cells, as outlined here for *SPAST* HSP. As discussed, it is expensive in time and money to generate iPS cells and this may be limiting for research on rare diseases like HSP. The strategy illustrated here, using two patient-derived stem cell models, olfactory neural stem cells and induced pluripotent stem cells, provides an efficient system to understand the disease mechanism at a cellular level and to develop therapeutics with the cheaper and easier ONS cells that are used for hypothesis generation and testing and iPS cell-derived neurons used for validation. With *SPAST* HSP, the most common form, as a starting point; the way is now open to emulate this research strategy for other forms. As knowledge builds for the effects of different mutations on cell phenotype, common and discrete disease mechanisms will be revealed and appropriate therapeutics can be devised.

## Figures and Tables

**Table 1:** Published studies of disease-associated cell deficits in *SPAST* patient-derived stem cell models and amelioration by tubulin-binding drugs.

Source of Patient-Derived Cells	Cell Model	Number of Cell Lines Used	Patient Cell Findings	Effects of Tubulin-Binding Drugs
Skin fibroblast cells [30]	iPS-derived glutamatergic neurons	2 patients/2 controls	Reduced spastin	-
			Increased p60 katanin	
			Reduced axon number, length and branching	
			Increased axonal swellings	
			Reduced mitochondria retrograde transport	
Skin fibroblast cells [29]	iPS-derived glutamatergic neurons	1 patient/1 control	Reduced spastin	Vinblastine reduced axonal swellings
			Increased stabilised microtubules	
			Increased axonal swellings	
			Reduced mitochondria retrograde transport	
Olfactory mucosa cells [61]	ONS	9 patients/10 controls	57% genes dysregulated	Taxol and vinblastine restored stabilised microtubules and cell size
			Reduced spastin	
			Reduced stabilised microtubules	
			Altered mitochondria and peroxisome distribution	
			Reduced peroxisome transport speed	
			Reduced cell size	
Olfactory mucosa cells [58]	ONS	9 patients/8 controls	Reduced stabilised microtubules	Taxol, vinblastine, noscapine and epothilone D restored stabilised microtubules and rescued peroxisome transport
			Reduced peroxisome transport speed	
Olfactory mucosa cells [62]	ONS and ONS-derived neuron-like cells	5 patients/5 controls	Altered peroxisome distribution	Epothilone D rescued increased vulnerability to oxidative stress
			Reduced microtubule-dependent peroxisome transport	
			Reduced retrograde peroxisome transport	
			Increased oxidative stress	
			Increased vulnerability to induced oxidative stress	

Abbreviations: iPS cells: induced Pluripotent Stem cells; ONS: Olfactory Neurosphere-derived Stem cells.
